# An Insight into the Anti-Angiogenic and Anti-Metastatic Effects of Oridonin: Current Knowledge and Future Potential

**DOI:** 10.3390/molecules26040775

**Published:** 2021-02-03

**Authors:** Nurul Akmaryanti Abdullah, Nur Fariesha Md Hashim, Aula Ammar, Noraina Muhamad Zakuan

**Affiliations:** 1 Department of Biomedical Sciences, Faculty of Medicine and Health Sciences, Universiti Putra Malaysia, Serdang 43400, Selangor, Malaysia; noraina@upm.edu.my; 2 Wolfson Wohl Translational Cancer Research Centre, Institute of Cancer Sciences, University of Glasgow, Bearsden, Glasgow City G61 1BD, UK; Aula.Ammar@glasgow.ac.uk

**Keywords:** oridonin, anti-angiogenic, anti-metastatic, angiogenesis, metastasis, invasion, migration

## Abstract

Cancer is one of the leading causes of death worldwide, with a mortality rate of more than 9 million deaths reported in 2018. Conventional anti-cancer therapy can greatly improve survival however treatment resistance is still a major problem especially in metastatic disease. Targeted anti-cancer therapy is increasingly used with conventional therapy to improve patients’ outcomes in advanced and metastatic tumors. However, due to the complexity of cancer biology and metastasis, it is urgent to develop new agents and evaluate the anti-cancer efficacy of available treatments. Many phytochemicals from medicinal plants have been reported to possess anti-cancer properties. One such compound is known as oridonin, a bioactive component of *Rabdosia rubescens*. Several studies have demonstrated that oridonin inhibits angiogenesis in various types of cancer, including breast, pancreatic, lung, colon and skin cancer. Oridonin’s anti-cancer effects are mediated through the modulation of several signaling pathways which include upregulation of oncogenes and pro-angiogenic growth factors. Furthermore, oridonin also inhibits cell migration, invasion and metastasis via suppressing epithelial-to-mesenchymal transition and blocking downstream signaling targets in the cancer metastasis process. This review summarizes the recent applications of oridonin as an anti-angiogenic and anti-metastatic drug both in vitro and in vivo, and its potential mechanisms of action.

## 1. Introduction

Cancer metastasis is considered the main cause of cancer mortality [1]. Even though metastatic cancer can be treated, this disease is generally considered incurable with a low survival rate [1,2]. Tumor metastasis is a dynamic multistep event that involves changes in various biochemical, genetic and epigenetic factors in the primary tumor that contributes to the invasion–metastasis cascade. This series of metastatic cascade begins with the invasion of the primary tumor cells into the surrounding tissues which is governed by the epithelial-to-mesenchymal transition (EMT) followed by the tumor cells entering the circulatory system and extravasating through the vascular walls into the parenchyma of distant tissues to form secondary cancers [3]. Tumor vascularization and angiogenesis are required for the dissemination and establishment of cancer metastasis [4]. According to Weinberg and Hanahan, inducing angiogenesis and the ability to invade surrounding tissues and metastasize are hallmarks of tumor malignancy [4].

New agents that present anti-cancer effects are largely tested for their ability to cause tumor shrinkage which focuses mainly on inhibiting cell division and proliferation. However, in advanced stages, cancer cells begin to invade the extracellular matrix (ECM), induce the EMT process, and create secondary tumors. The current cancer treatment strategies such as chemotherapy are often associated with adverse side effects and greatly affects the quality of life. Moreover, treatment resistance is also often observed when cancer is presented at an advanced stage [5]. It is therefore important to vigorously identify new agents with low susceptibility to drug resistance and to explicitly block key molecules or their downstream signaling targets in the cancer metastasis process.

**Figure 1:**
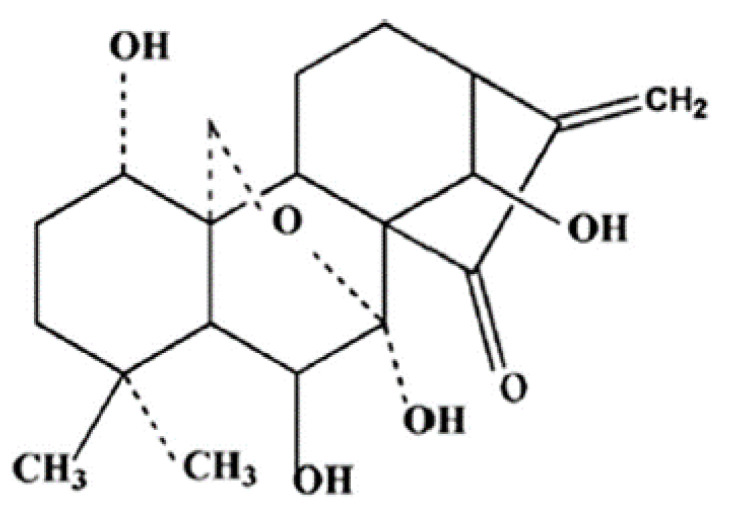
The chemical structure of oridonin.

Natural products derived from Chinese medicinal plants have recently attracted a growing interest in the treatment of cancer, especially those with low side effects such as shikonin, berberine, and curcumin [6]. In China, *Rabdosia rubescens* is used as an alternative medicine to treat sore throat, gingivitis, and rheumatoid arthritis [7,8]. Oridonin (C_20_H_28_O_6)_ (Figure 1) is an active diterpenoid component from *Rabdosia rubescens* first identified in 1966 by Furida and colleagues [9]. Oridonin possesses many therapeutic potentials such as neuroprotective [10], anti-inflammatory [11] and antibacterial [12] effects. In recent years, the anti-cancer properties of oridonin were studied in a wide range of tumors including breast [13], colon [14], pancreatic [15], lung [16], gastric [17], prostate [18] and skin [19] cancer. This review discusses the different molecular pathways involved in angiogenesis, cell invasion, and metastasis that can be targeted by oridonin.

## 2. The Process of Angiogenesis as a Target of Oridonin

Tumor angiogenesis is a highly regulated process through which new blood vessels form in the tumor environment to supply oxygen and support tumor growth [20]. In this process, extensive interplays between endothelial cells, angiogenic growth factors, and ECM is required [20,21]. In tumor angiogenesis, various pro-angiogenic signals such as vascular endothelial growth factor (VEGF), epidermal growth factor (EGF), and transforming growth factor (TGF) are released to induce the formation of blood vessels and to support tumor proliferation [20,22]. Such a process is also referred to as the ‘angiogenic switch’ and can occur at different stages of tumor progression as a result of genetic or microenvironmental changes [23]. During the switch, tumors in dormancy re-enter the cell cycle and become actively growing malignant cells [23,24]. One of the angiogenic models includes new vessels sprouting from pre-existing ones in the tumor microenvironment [25]. In this process, endothelial cells emerge towards an angiogenic stimulus, and endothelial cells involved are classified into tip and stalk cells [25]. The tip cells are responsible for ECM degradation while the stalk cells facilitate vascular lumen formation [26,27]. During sprouting angiogenesis, VEGF and Notch signaling are activated to guide the vascular patterning by directing the tip cell migration and stalk cell proliferation. VEGF can induce jagged ligands which then increases Notch expression in cancer endothelial cells to promote Notch-dependent angiogenesis [28]. VEGF also activates other signaling cascades such as phosphatidylinositol 3-kinase (PI3K)/Akt and mitogen-activated protein kinase. After the establishment of new blood vessels, platelet-derived growth factor β (PDGF) is secreted to assist the maturation process of blood vessels [25]. In addition to sprouting angiogenesis, cancer cells use vascular mimicry to acquire blood supply [29]. Highly invasive tumors can differentiate into endothelial cells and induced tube-like structures. This allows tumors to have a secondary circulatory system that is not dependent on angiogenesis [29].

Based on the understanding of the diverse pathways involved in angiogenesis, strategies to inhibit the formation of new blood vessels in tumors can be applied at different stages such as targeting the proangiogenic factors or disrupt the active dividing endothelial cells. Over the years, many anti-angiogenic inhibitors have been developed such as bevacizumab [30] and sunitinib which target VEGF pathways [31]. VEGF is one of the most extensively studied angiogenic factors and a key mediator in tumor angiogenesis. The VEGF family has at least seven isoforms (i.e., VEGF-A, VEGF-B, VEGF-C, VEGF-D, VEGF-E, placental growth factor, and VEGF-F) that bind to the tyrosine kinase receptor known as vascular endothelial growth factor receptor (VEGFR) [32]. High expression of VEGF has been reported in many cancers such as breast [33], prostate [34], and ovarian [35] cancer.

Targeting angiogenesis has so far shown limited success, which may be attributed to the heterogeneity of blood vessels in the tumor environment and tumor hypoxia [20]. The newly formed blood vessel in tumor is usually abnormal in structure with immature and leaky blood vessels. Unlike normal blood vessels, the tumor vessels are narrower in diameter, have diverse vessel density, and high permeability [36]. Moreover, tumor blood vessels are not efficient in delivering oxygen and removal of waste products which can lead to an aggressive tumor microenvironment [20]. As a result of the abnormality of tumor blood vessels, the efficacy of anti-cancer treatment may be decreased due to the inaccessibility of the drug to the tumor area and increased drug resistance [25]. The development of new anti-angiogenic agents with high efficacy and fewer side effects is needed to overcome resistance to the existing agents and improve cancer therapy.

In recent years, oridonin has been shown to be a promising anti-angiogenic agent. Dong and coworkers reported that oridonin inhibits angiogenesis by blocking VEGF-induced micro-vessels sprouting. In human umbilical vein endothelial cells (HUVEC) cells, oridonin treatment reduced more than 90% tubular formation [37]. In another study, Jiang and colleagues reported that VEGF-induced migration was reduced following oridonin treatment. The tubular formation was also decreased by 70% in the oridonin treated group when compared to the control group. Also, oridonin treatment has resulted in irregular and disorganized tube formation and the depolymerization of F-actin [38]. Vascular assay in zebrafish embryos showed that oridonin reduced the diameter of the complete intersegment vessels when compared to the control group [39]. Taken together, these suggest that oridonin may interfere with capillary network formation and actin organization.

The key targets of oridonin to suppress angiogenesis include VEGF, Notch, and PI3K signaling pathways. Oridonin has been shown to inhibit the expression of the VEGF family such as VEGF-A, VEGFR-2, and VEGFR-3 [39,40,41]. In endothelial cells, when VEGF binds to its receptor, the VEGF/PI3K signaling pathway is activated to induce vessel formation [42]. Oridonin also inhibited the VEGF-induced Notch activation by reducing the expression of key ligand and downstream genes including Jagged-1 and -2, Notch 1, Hes-1, HESR-1, and DII-1 [37]. The inhibition of VEGF expression by oridonin ultimately leads to the suppression of angiogenesis due to the inactivation of its downstream targets.

**Figure 2:**
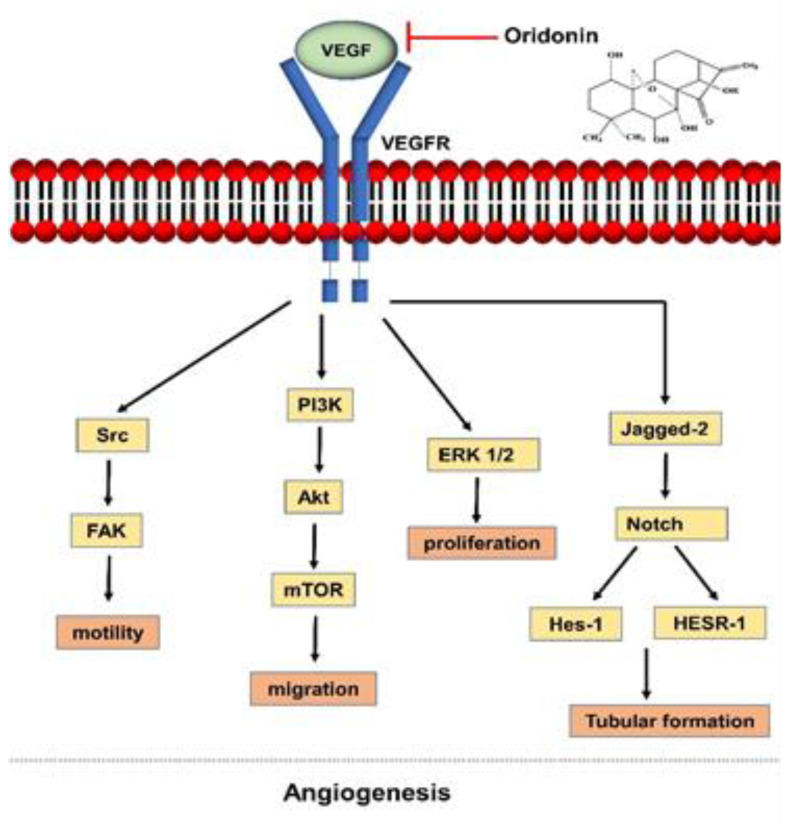
The effects of oridonin on angiogenesis. Oridonin inhibits angiogenesis by blocking the activation of VEGF and its downstream signaling pathways such as Src, PI3K, ERK 1/2, and Notch in endothelial cells. Abbreviations: vascular endothelial growth factor (VEGF); vascular endothelial growth factor receptor (VEGFR); focal adhesion kinase (FAK); phosphatidylinositol 3-kinase (PI3K), mammalian target of rapamycin (mTOR); extracellular signal-regulated protein kinase (ERK).

The combination treatment of chemotherapy agents with anti-angiogenic inhibitors has been shown to improve treatment efficacy in ovarian cancer. For example, the administration of bevacizumab with selected chemotherapy agents such as pegylated liposomal doxorubicin and paclitaxel improved progression-free survival in platinum-resistant ovarian cancer [43]. Li and colleagues explored the potential of an anti-angiogenesis effect of oridonin in combination with doxorubicin [44]. Treatment with doxorubicin alone did not inhibit cell migration and invasion of HUVECS cells. However, oridonin treatment as a single agent resulted in reduced VEGF-induced endothelial cell migration and tube formation. Interestingly, the combination of both compounds synergistically impedes cell migration and invasion of HUVECS cells. Based on the molecular docking study, a combination of the two compounds showed a high affinity towards the ATP-binding domain of VEGFR-2 kinase which suggests that the interaction may inhibit the activation of VEGFR-2 [44]. Figure 2 summarizes the effect of oridonin on angiogenesis.

## 3. Oridonin in EMT

EMT is a complex biological process in which epithelial transitions into a mesenchymal phenotype and it is one of the important events in driving tumor progression and metastasis [45]. In EMT, epithelial cells lose their epithelial characteristics and gain motile mesenchymal properties such as loss of cell-cell adhesion, augmented motility, and invasiveness [46]. The shift from one state to another is controlled by a range of growth factors and signaling pathways [47]. The strong integration between these growth factors such as TGF-β forms a robust network promoting the growth of cancer cells and that makes EMT a possible target for cancer metastases. There are various ways to target the EMT process in cancer which include (i) blocking the activation signal of EMT markers such as E-cadherin, N-cadherin vimentin, fibronectin, matrix metalloproteinases (MMPs), and TGF-β [48], (ii) reversed the EMT process, and (iii) suppressing the growth EMT-like cells. E-cadherin and N-cadherin share a similar structure and are involved in cell-cell adhesion [46]. On the other hand, vimentin and fibronectin play a role in maintaining cell shape and cell adhesion, respectively [49,50]. The reduced expression of E-cadherin along with the concomitant increased expression of specific mesenchymal markers such as zinc finger E-box binding homeobox 1 (ZEB1), N-cadherin, and vimentin are considered hallmarks of EMT [46,51]. Growing evidence showed that oridonin exhibits anti-metastatic effects by altering the EMT pathway. To date, the effects of oridonin on the EMT pathways have been reported in many cancers such as pancreatic, breast, melanoma, and lung cancer. Previous studies suggest that oridonin increased E-cadherin expression while decreased the expression of ZEB1, N-cadherin, fibronectin, vimentin, snail, and slug [52,53,54]. The MMPs play a pivotal role in cancer cell invasiveness and metastasis by facilitating ECM degradation [55]. As an invasion promoter, MMPs can facilitate EMT through invasion and metastasis behaviors (i.e., regulation of actin cytoskeleton, increase motility, and proliferation) [56]. Various studies demonstrated that oridonin could inhibit the expression of MMP-2 and MMP-9 in various cancers such as breast [13], acute myeloid leukemia [57] bone [58], and ovarian [59] cancer. Oridonin also decreased MMP-12 expression which is an important mediator to degrade ECM in lung cancer [60]. The effects of oridonin on EMT makers and regulators are summarized in Table 1.

## 4. Cancer Invasion and Metastasis

**Figure 3:**
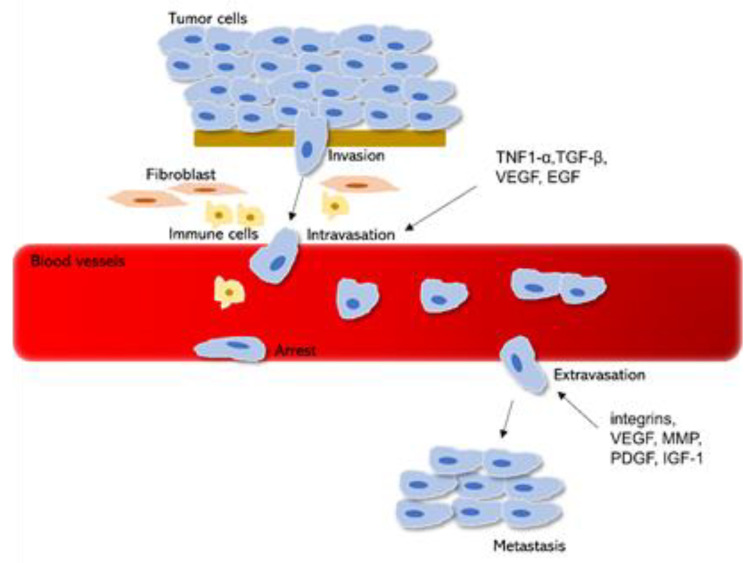
Overview of the metastasis process from primary tumor cells intravasate into the blood circulation followed by extravasation, supported by various growth factors, and settlement of the cancer cells at a distinct tissue site. Abbreviations: tumor necrosis factor 1α (TNF1-α); transforming growth factor- β (TGF-β),vascular endothelial growth factor (VEGF); epidermal growth factor (EGF); matrix metalloproteinase (MMP); platelet-derived growth factor (PDGF); insulin-like growth factor-1 (IGF-1).

The invasion-metastatic pathway is a multi-step process that starts with the invasion of the cancer cells into the surrounding tissues (Figure 3). In this process, the release of MMPs, hyaluronidase, and metalloproteinase (ADAM) are required to assist ECM degradation and remodeling [63]. Following ECM degradation, tumor cells then migrate into the blood and lymphatic vessels crossing the endothelial cell barrier and this step is known as intravasation. Cancer cells escape into the circulation either as single circulating tumor cells (CTC) or as clustered CTC. To promote cancer cells transmigration into the circulatory system and protect them from external insults, various growth factors and cytokines are released including tumor necrosis factor 1-α (TNF1-α), TGF-β, VEGF, and EGF [3]. The release of EGF and TGF-β enhances the intravasation process by allowing the cancer cells to cross blood vessels barriers [64]. To establish a secondary tumor at a distant tissue site, cancer cells need to extravasate from the vascular system. The releases of integrins, insulin-like growth factor-1 (IGF-1), VEGF, MMP, PDGF, and help to facilitate cancer cell extravasation [3] (Figure 3). Integrins support the anchorage-independent survival of CTC while VEGF and IGF1 induce vascular permeability to allow them to penetrate endothelial cells and migrate into tissue parenchyma [65]. Metastatic colonization begins once cancer cells survive the stressful processes of intravasation and extravasation. In this stage, cancer cells can either proliferate continuously or enter dormancy [66]. The tumor microenvironment at the metastatic site plays a key role in determining whether cancer cells can survive. Factors such as favorable conditions in ECM, effective vascular system, ability to escape immune system surveillance, and resistance to anoikis will determine whether cancer cells enter dormancy or proliferate into macrometastases [66].

Limited progress has been made in metastatic cancer therapy due to multiple factors such as tumor growth in different organs, the complexity of metastatic cascade, and increased resistance to cytotoxic agents [45]. Furthermore, the survival of patients with metastatic cancer remains poor [2]. Several strategies have been identified to target metastatic cascades. These include targeting the early steps in the metastatic processes, prevent tumor dissemination into the circulatory system, and killing dormant cancer cells [65]. Over the years, various inhibitors targeting key molecules in cancer metastasis have been developed such as MMP, VEGF, and endothelial growth factor receptor (EGFR) inhibitors [65,67]. However, many of these agents failed to show efficacy and safety in clinical trials [68,69]. Identification of an agent that can inhibit a target integral to multiple stages of metastasis can significantly enhance metastasis inhibition and increase patient survival. Oridonin has been reported to exhibit an anti-metastatic effect in cancer. Oridonin inhibits cancer cells invasion and metastasis through targeting various transcription factors and their signaling pathways such as TGF-β1 [70], EGFR [60], mammalian target of rapamycin (mTOR) [71], long non-coding RNAs (lncRNAs) [53] as well as increasing the activity of tumor suppressor genes (i.e., p53 and protein phosphatase 2A (PP2A)) [72]. The effects of oridonin on these targets are discussed below.

### 4.1. Oridonin Inhibits TGF-β/Smad Pathway

In human cancers, dysregulation of TGF-β is common in many human cancers such as prostate and bone cancer [73,74]. In the early stage of cancer development, TGF-β acts as a tumor suppressor, but as cancer progresses TGF-β enhances cancer cell invasiveness and increases metastases [75]. Smad is activated by phosphorylation via TGF-β1, translocates to the nucleus where it forms a complex that works as a transcription factor and triggers a cascade of gene expression involved in cell migration such as integrins and MMPs [76,77]. In osteosarcoma, 24 h treatment with low concentrations of oridonin (0.5–2 µM) decreased the migration and invasion capabilities of 143B and MG-63 bone cancer cells by significantly inhibiting the phosphorylation of TGF-β and preventing the Smad dimer from translocating into the nucleus [54]. Bu and coworkers also demonstrated that oridonin blocks the activation of the TGF-β1/Smad signaling pathway in colon cancer. In this study, pre-treatment with 8 µg/mL of oridonin for 48 h downregulated the phosphorylation of TGF-β1 downstream effectors like Smad proteins (Smad2, Smad3, and Smad4) and subsequently deactivated plasminogen activator inhibitor type 1 (PAI-1), a molecule regulated by Smad pathway [70].

Oridonin was shown to inhibit the cellular migration and invasion and decrease the number of cells adhesiveness to fibronectin in B16-F10 (mouse) and A375 melanoma cancer (human) cells via downregulation of TGF-β1 expression and inhibition of the PI3K/Akt/GSK-3β signaling pathway [78]. In a recent study, treatment with oridonin (4–16 µM) for 24 h inhibited regulatory T cell (Treg) differentiation in 4T1 murine breast cancer cells via downregulation of the protein levels of TGF-β receptor, Smad2, and Smad3. In vivo study also demonstrated that oridonin at doses of 2.5, 5 and 10 mg/kg reduced Treg phosphorylation thereby suppressing breast cancer growth and progression [79]. Such data suggest that oridonin exhibits an anti-metastatic effect by inhibits TGF-β/Smad pathway.

### 4.2. Oridonin Blocks the Activation of EGF/EGFR/ERK Signaling Pathways

High expression of EGFR was observed in many tumors such as in lung, breast, and ovarian cancer [80,81]. The aberration in EGFR expression and downstream signaling influenced tumor progression and maintenance of the malignant phenotype [82]. Phosphorylation of EGFR activates the extracellular-signal-regulated kinase (ERK) pathway that is involved in various pathological processes including angiogenesis, migration, and invasion. In lung cancer, treatment with oridonin (0–10 µM) for 24 h suppressed cell migration, invasion, and adhesion of H1975 human non-small cell lung cancer (NSCLC) cell line through suppression of phosphorylation of EGFR and its downstream signaling pathway via ERK [60]. Protein phosphatase 2A (PP2A) is a tumor suppressor gene that inactivates the ERK pathway [83]. Xiao and colleagues demonstrated that oridonin (0–10 µM) increased PP2A activity and inactivated the ERK/Akt pathway which resulted in inhibition of cell migration and invasion of H1975 NSCLC cells after 24 h treatment. The inhibition of the ERK/Akt pathway also promoted apoptosis in these cells [60]. Previous studies have reported that in transformed cells and cancer cell lines, inhibition of PP2A promotes cell motility [84,85]. The activation of the ERK pathway is also mediated by focal adhesion kinase (FAK) [86]. The activation of FAK can lead to increased motility of cancer cells. Wang and colleagues showed that treatment with oridonin (1.25–5 µM) for 24 h inhibits MDA-MB-231 breast cancer cell motility and migration activities via suppression of FAK and integrin β1 expressions [13]. In H1688 NSCLC cell line, oridonin at a concentration of 10 µM markedly inhibited cell migration via downregulating the expression of metalloproteinases and phosphorylated FAK (p-FAK) [61]. The inhibition of EGF/EGFR signaling pathway by oridonin results in the suppression of ERK and FAK leading to a decrease in cell motility, migration, and invasion capacities of cancer cells.

### 4.3. Oridonin Inhibits the Phosphorylation of mTOR Signaling Pathway

Growing evidence supports the role of mTOR is in cancer cell invasion and metastasis especially by regulating the organization of actin cytoskeleton [87,88]. The anti-invasive and anti-metastatic effects of oridonin via inhibiting the mTOR pathways have been studied in ovarian cancer. The treatment with oridonin at 2.5 to 10 µM for 24 h decreased cell migration and invasion of SKOV3 ovarian cancer cells by blocking the phosphorylation of the mTOR signaling pathway [71,89]. The inhibition of mTOR is also accompanied by the upregulation of forkhead box P3 (FOXP3) following oridonin treatment [89]. FOXP3 plays a role in regulating the function of regulatory T-cell [90]. Moreover, FOXP3 was also reported to play role in cancer metastasis [91]. Previous studies also showed that the inhibition of mTOR by oridonin induced cell apoptosis [92,93]. Such findings suggest that the inhibition of mTOR signaling pathways and the involvement of FOXP3 are essential for the anti-tumorous effect of oridonin.

### 4.4. Oridonin Downregulates the lncRNA AFAP1-AS1 Expression

The long non-coding RNAs (lncRNAs) are involved in various biological processes including cell differentiation, proliferation, growth, and apoptosis [94]. RNA actin filament-associated protein 1 antisense RNA 1 (AFAP1-AS1) is a recently identified cancer-associated lncRNA originating from the antisense DNA strand of the AFAP1 coding gene locus. The aberrant expression of lncRNAs is frequently reported in cancer [95]. In pancreatic cancer, overexpression of lncRNA AFAP1-AS1 is associated with low survival and disease progression [96]. As a result, lncRNA AFAP1-AS1 has become a possible target in the treatment of pancreatic cancer. A recent study has shown that treatment with oridonin (95 µM) and/or knockdown of lncRNAs AFAP1-AS1 for 24 h inhibited cell invasion capacity of PANC-1 and BxPC-3 pancreatic cancer cell lines as assessed by transwell migration assay. In lncRNA AFAP1-AS1 knockdown cells, oridonin treatment resulted in fewer cells penetrating through the membranes in transwell assays compared to siAFAP1-AS1 alone. The author suggested that oridonin maintained AFAP1-AS1 inhibition which further decreased the metastasis activity of PANC-1 and BxPC-3 cells [53]. This indicates that oridonin increases its anti-metastatic effect against pancreatic cancer by continuously inhibiting AFAP1-AS1.

### 4.5. Oridonin Increases the Expression of p53

Any mutations or loss in the p53 tumor suppressor gene can result in uncontrolled cell division, avoidance of apoptosis, and changes in cell migration and polarity [97]. Studies have shown that loss of p53 function is associated with an increase in cell motility and hence facilitates cancer development and metastases [97,98]. The p53 level and activity are mainly mediated by ubiquitin E3 ligase Mdm2, which binds directly to p53 and facilitates p53 ubiquitination and proteasomal degradation [99]. Mdm2 is known as the principal negative regulator of p53 [99]. Treatment with oridonin (10–80 µM) for 24 h was shown to suppress the migration activity of SNU-16 gastric cancer cells via apoptosis, increase the expression of p53, and downregulate the expression of Mdm2. The inhibitory effect of oridonin was reversed in a stable knockdown of p53 by siRNA in SNU-16 cells validating the above observation [72]. A previous study reported that p53 mutations occur at a late stage in cancer progression pathway in 56% of gastric carcinoma cases [100]. The mutational spectrum of p53 in gastric cancer is wide however, the most common sites occur at codon 175, 213, 245, 248, 273, and 282 [101]. An increase in p53 level and activity following oridonin treatment (20–40 µM) was also observed in other cancer such as neuroblastoma [102] and esophageal [103] cancer. Taken together, these data propose that oridonin exert its anti-cancer activity by enhancing p53 protein expression in cancer cells and promotes apoptosis.

## 5. Oridonin in Hypoxia

Hypoxia is the main feature of solid tumors and it arises as a result of uncontrolled proliferation of cancer cells which limit the availability of oxygen supply and, often associated with poor overall survival [104,105]. The unorganized vascular networks at the tumor site make a significant contribution to a reduced level of oxygen in the solid tumor [104]. In metastatic cancer, hypoxia serves as one of the main drivers for cancer to develop a more aggressive and resistant phenotype as well as enhanced survival in a nutrient-deprived environment [106,107]. This is because hypoxia increases the expression of many angiogenic inflammatory markers and growth factors in cancer cells particularly VEGF-A [108]. Besides, hypoxia may induce EMT by promoting the transcription of EMT markers such as Snail, ZEB1, and TWIST [107]. The activation of hypoxia-induced EMT markers promotes cancer cell motility, migration, and invasion, resulting in tumor progression and metastases [109]. Hypoxia-inducible factor 1 (HIF-1α) is a major transcriptional regulator in hypoxia which can directly or indirectly regulate EMT markers [109]. For instance, HIF-1α may activate N-cadherin and vimentin to mediate EMT by promoting the loss of cell-cell adhesion which subsequently results in more migratory and invasive cancer cells. HIF-1α has been considered a therapeutic target for the treatment of cancer metastasis by inhibiting HIF-1 and its downstream molecules using bioreductive drugs and gene therapy [110]. Bioreductive drugs are inactive agents that undergo biotransformation to generate highly reactive electrophiles through enzymatic reduction catalyzed by endogenous oxidoreductases. The activation of such agents occurs in hypoxic regions where oxygen levels are low [111]. Examples of these agents include tirapazamine, mitomycin C and E09. These agents, however, showed limited success in clinical trials due to toxicity and rapid clearance [112,113]. Therefore, it is important to identify new agents that can target hypoxia without inducing severe toxicity. Oridonin has been shown to inhibit hypoxia-induced migration and EMT via targeting HIF-1α. In human gallbladder cancer cells (GBC-SD), treatment with 5 µM of oridonin for 24 h significantly inhibited EMT and reversed hypoxia-induced migration via downregulation of HIF-1α/MMP-9 signaling pathways. Similarly, in tumor xenograft tissue, treatment with 5 µM of oridonin suppressed the protein expression of HIF-1α and MMP-9 [114]. In MDA-MB-231 and 4T1 breast cancer cells, treatment with increasing concentration of oridonin (0–16 µM) for 24 h inhibited angiogenesis and cell migration via downregulation of HIF-1α protein expression [40]. Taken together, these findings suggest that, through downregulation of HIF-1α protein expression, oridonin is able to suppress cell migration, angiogenesis and EMT.

## 6. Conclusions and Future Perspectives

Disturbance of metastases processes carries a great amount of clinical significance for patients with or at risk of developing metastatic cancer. Herbal medicines have long been an essential source for the discovery and development of new drugs against human diseases. The use of natural products in the treatment of cancer has gained the attention of the research community due to low cost and few side effects. In this review, we have summarized many potential therapeutic advantages of oridonin particularly in targeting angiogenesis and metastasis. The anti-angiogenic effect of oridonin is observed in its ability to target VEGF and suppress the formation of blood vessels. Moreover, when combined with other chemotherapy drugs such as doxorubicin, the effectiveness of treatment is improved. The ability of oridonin to interrupt several metastasis pathways holds preclinical promise for its development as a potential anti-metastatic agent in clinical settings. Oridonin has been shown to inhibit cell migration and invasion by targeting several metastatic signaling pathways. However, as tumor metastasis is a complex disease, more studies on other metastatic pathways should be considered. Over the years, researchers also have developed novel oridonin analogs such as HAO472 [115], CYD-6-17 [116], 1-O- and 14-O-derivative compounds [117]. The development of these novel analogs is aimed to improve oridonin’s water solubility and therapeutic efficacy. Many of these agents, however, have not been tested for their anti-angiogenic and anti-metastatic effects. Additional research is warranted to explore the potential therapeutic benefits of oridonin and its derivatives in the management of metastatic cancer especially in animal models and clinical trials.

## Figures and Tables

**Table 1:** Effects of EMT markers modulated by oridonin.

EMT Marker	Cancer Type	Cell Line	Concentration and Treatment Time	Effect	References
ZEB1	Pancreatic	BxPC-3	87.8–95 µM (24 h)	Downregulation of ZEB1 protein expression	[52,53]
		PANC-1	55.8 μM–95 µM (24 h)		
E-cadherin	Pancreatic	BxPC-3	95 µM (24 h)	Enhances of E-cadherin protein expression	[53]
		PANC-1	95 µM (24 h)		
		SW1990	15 µM (24 h)	Enhances of E-cadherin protein expression	[61]
	Breast	MDA-MB-231	4–16 µM (24 h)	Upregulation of E-cadherin mRNA and protein expression	[40]
		4T1			
	Bone	MG-63	0.8–2 µM (24 h)	Upregulation of E-cadherin mRNA and protein expression	[54]
		143B			
	Lung	H1688	5–10 µM (24 h)	Upregulation of E-cadherin mRNA and protein expression	[16]
N-cadherin	Pancreatic	BxPC-3	87.8–95 µM (24 h)	Downregulation of N-cadherin protein expression	[52,53]
		PANC-1	55.8 μM–95 µM (24 h)		
	Breast	MDA-MB-231	4–16 µM (24 h)	Downregulation of N-cadherin mRNA and protein expression	[40]
		4T1			
	Bone	MG-63	0.8–2 µM (24 h)	Downregulation N-cadherin mRNA and protein expression	[54]
		143B			
Fibronectin	Pancreatic	BxPC-3	87.8 µM (24 h)	Downregulation of fibronectin protein expression	[52]
		PANC-1	55.8 μM (24 h)		
Vimentin	Breast	MDA-MB-231	4–16 µM (24 h)	Downregulation of vimentin mRNA and protein expression	[40]
		4T1			
	Pancreatic	SW1990	15 µM (24 h)	Downregulation of vimentin mRNA levels	[61]
	Bone	MG-63	0.8–2 µM (24 h)	Downregulation of vimentin mRNA and protein expression	[54]
		143B			
	Lung	H1688	5–10 µM (24 h)	Downregulation of vimentin mRNA and protein expression	[16]
Snail	Breast	MDA-MB-231	4–16 µM (24 h)	Downregulation of snail mRNA and protein expression	[40]
		4T1			
	Pancreatic	SW1990	15 µM (24 h)	Decreases snail mRNA levels	[61]
		BxPC-3	95 µM (24 h)	Downregulation of snail protein expression	[53]
		PANC-1			
	Bone	MG-63	0.8–2 µM (24 h)	Downregulation of snail mRNA and protein expression	[54]
		143B			
	Lung	H1688	5–10 µM (24 h)	Downregulation of snail mRNA and protein expression	[16]
	Skin	A375	20 µM (12 h)	Downregulation of snail protein expression	[62]
		MDA-MB-435S			
Slug	Pancreatic	SW1990	15 µM (24 h)	Decreases slug mRNA levels	[61]
		BxPC-3	95 µM (24 h)	Downregulation of slug protein expression	[53]
		PANC-1			
	Bone	MG-63	0.8–2 µM (24 h)	Downregulation of slug mRNA and protein expression	[54]
		143B			
	Lung	H1688	5–10 µM (24 h)	Downregulation of slug mRNA and protein expression	[16]
MMP-2	Breast	MDA-MB-231	1–5 µM (24 h)	Downregulation of MMP-2 protein expression	[13]
	AML	MV4-11/DDP	10–80 µM (48 h)	Downregulation of MMP-2 protein expression	[59]
	Bone	U2OS	15–60 µM (48 h)	Downregulation of MMP-2 protein expression	[58]
	Ovarian	A2780/DDP	10–80 µM (48 h)	Downregulation of MMP-2 protein expression	[57]
MMP-3	Bone	U2OS	15–60 µM (48 h)	Downregulation of MMP-3 protein expression	[58]
MMP-9	Breast	MDA-MB-231	1–5 µM (24 h)	Downregulation of MMP-9 protein expression	[13]
	AML	MV4-11/DDP	10–80 µM (48 h)	Downregulation of MMP-9 protein expression	[59]
	Bone	U2OS	15–60 µM (48 h)	Downregulation of MMP-9 protein expression	[58]
	Ovarian	A2780/DDP	10–80 µM (48 h)	Downregulation of MMP-9 protein expression	[57]
MMP-12	Lung	H1975	10–20 µM (24 h)	Downregulation of MMP-12 protein expression	[60]

Abbreviations: epithelial-to-mesenchymal transition (EMT); zinc finger E-box binding homeobox 1 (ZEB1), matrix metalloproteinases (MMP); acute myeloid leukemia (AML); messenger RNA (mRNA).

## Data Availability

Data sharing not applicable.

## Abbreviations

EMT	epithelial-to-mesenchymal transition
ECM	extracellular matrix
VEGF	vascular endothelial growth factor
EGF	epidermal growth factor
TGF	transforming growth factor
VEGFR	vascular endothelial growth factor receptor
MMP	matrix metalloproteinases
ZEB1	zinc finger E-box binding homeobox 1
ADAM	metalloproteinase
CTC	circulating tumor cells
TNF1-α	tumor necrosis factor 1-α
PDGF	platelet-derived growth factor
IGF-1	insulin-like growth factor-1
lncRNAs	long non-coding RNAs
EGFR	endothelial growth factor receptor
mTOR	mammalian target of rapamycin
NSCLC	human non-small cell lung cancer
ERK	extracellular-signal-regulated kinase
PP2A	protein phosphatase 2A
FAK	focal adhesion kinase
FOXP3	forkhead box P3
HIF-1α	hypoxia-inducible factor 1
